# Development of a system to support information sharing for medical staff in the hybrid emergency room

**DOI:** 10.1002/ams2.70006

**Published:** 2024-09-20

**Authors:** Shuhei Maruyama, Yasushi Nakamori, Shuji Kanayama, Daiki Wada, Fukuki Saito, Yasuyuki Kuwagata

**Affiliations:** ^1^ Department of Emergency and Critical Care Medicine Kansai Medical University Medical Center Moriguchi Osaka Japan; ^2^ Department of Emergency and Critical Care Medicine Kansai Medical University Hospital Hirakata Osaka Japan

To the Editor,

The hybrid ER^1^, ^2^, ^3^ can significantly reduce time required for initial emergency care, but extensive information gathered during treatment needs to be interpreted and reflected in the treatment strategy within a short period of time. We report on the development of an information sharing system for initial emergency care.

During initial emergency care, vital signs, blood gas analysis (BGA), and laboratory data are important factors in decision making. When we perform surgery or transcatheter arterial embolization (TAE), knowing the trend of each parameter is important in determining whether vital signs have stabilized or worsened as a result of the therapeutic intervention. Vital sign monitors provide real‐time vital signs but no information on their trend. In contrast, information systems commonly used in the intensive care unit or operating room provide trend information, but they update at a minimum of every minute. Updating at a minimum of every minute is too long for initial emergency care in which the critical patient's condition is constantly changing. Our developed system displays trend information on heart rate, blood pressure, and BGA that updates every 5 s and shows their trends over 180 min. Medical staff may not notice sudden changes in vital signs or results of BGA or laboratory data. When vital signs exceed a predetermined threshold, the system alerts them through an audio signal in the ER and in headsets and provides specific values for the medical staff. When updated results of BGA and laboratory tests are received, they are automatically and promptly showed in the monitor and read out as well. The manager could set any items in BGA and laboratory data to be read out by this system to their liking (e.g., calcium ions, CO‐Hb). This allows the staff to recognize changes in vital signs and test results with no time lag. Initial emergency care, especially in trauma patients, is always a race against time. The system displays the time since the start of monitoring, surgery, TAE, and aortic blockade in a count‐up format and alerts the medical staff via audio of the time that has elapsed. The system has two modes: standard mode and critical care specific (Figure 1 and Videos [Link], [Link]). It responds to requests input through icons on the touch screen or by voice. This interactive utility is used to record various procedures, start and end times of measurements, and reconfirm information. Monitoring the administration of blood transfusion could be difficult in the emergency setting. The system displays administered transfusions (e.g., RBC, FFP, PC and cryoprecipitate) as the icons on the monitor manual input. In this way, the changes in vital signs and laboratory tests due to transfusion could be recognized on the screen. Dynamic monitoring updated every 5 s of vital signs are displayed on a 55‐inch monitor so that staff can check the trend at any time and be alerted to changes in the patient's condition. We believe this system to be highly useful in improving functionality of the hybrid ER and enhancing the rate of life saving.

**FIGURE 1:**
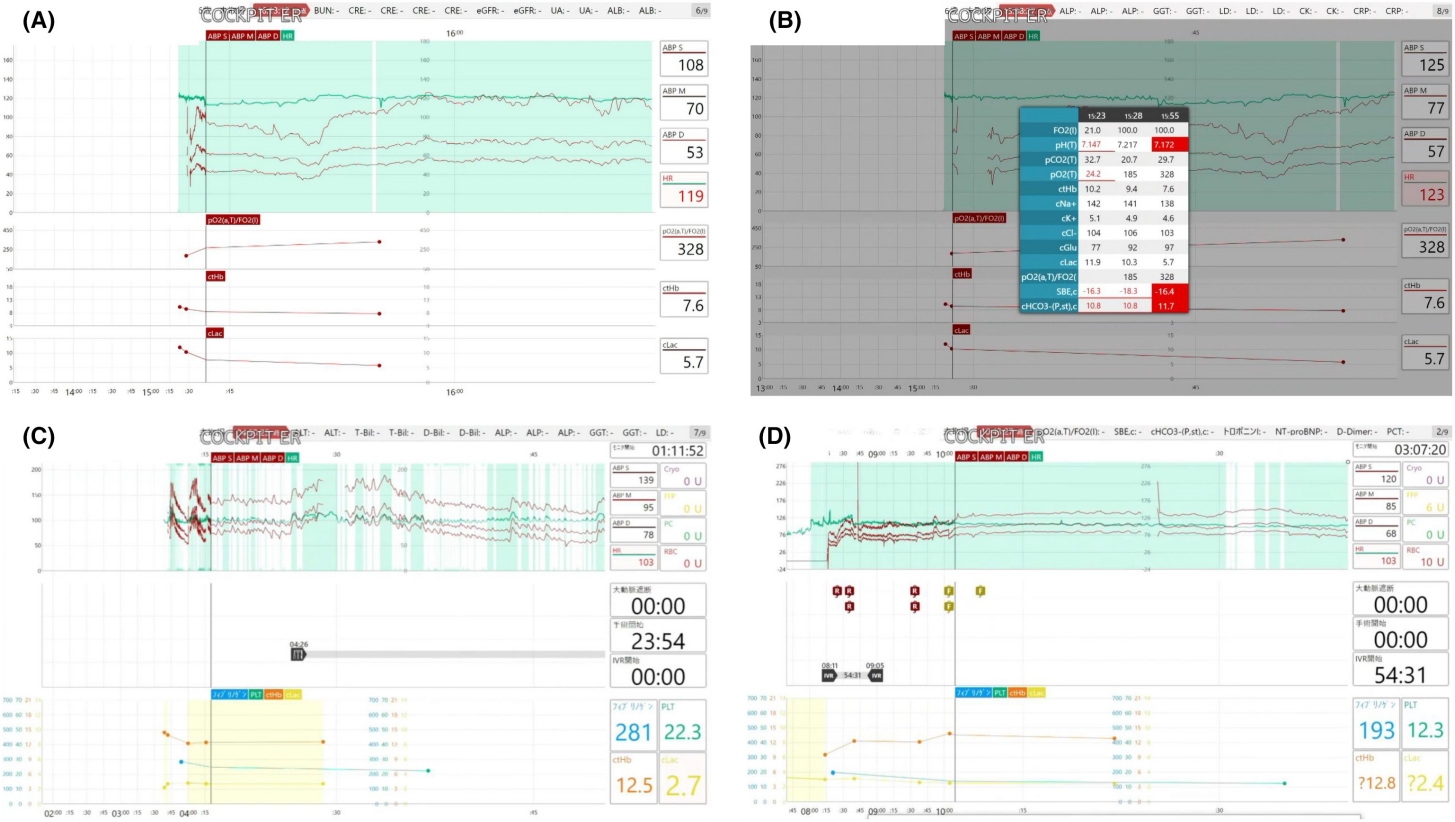
Monitor screen of this system. (A) This screen shows standard monitoring mode in which the upper panel reveals the trend in the vital signs (heart rate, blood pressure), and the lower panel reveals the trend in blood gas analysis data (PF ratio, hemoglobin, and lactate). Time is on the horizontal axis, with the rightmost point being the present time and the leftmost point being 180 min ago. When patient monitoring is started, the waveform flows from right to left. (B) When the results of BGA and laboratory data are obtained, they are displayed on the monitor and read out in the ER and through headsets. (C, D) Critical care‐specific mode. The upper panel displays vital signs, the middle panel displays information about transfusions, procedures (surgery, TAE, aortic blockade, etc.), and the bottom panel displays trends in laboratory results (hemoglobin, lactate, platelet count, and fibrinogen). On the far right, respective times (time after patient transfer, start of surgery, TAE, and aortic blockade) are displayed in a count‐up fashion. Similarly, total transfusion volume is also reflected.

## CONFLICT OF INTEREST STATEMENT

Shuhei Maruyama received lecture fees from Canon Medical Systems Co. Dr. Yasushi Nakamori received research grants from Canon Medical Systems Co. Dr. Yasuyuki Kuwagata is an Editorial Board member of the *Acute Medicine & Surgery* journal and a coauthor of this article. To minimize bias, he was excluded from all editorial decision‐making related to the acceptance of this article for publication.

## ETHICS STATEMENT

Approval of the research protocol: This study was conducted according to the principles of the Declaration of Helsinki and approved by Kansai Medical University Medical Center Institutional Review Board (Study Number: 2023283).

Informed consent: N/A.

Registry and the registration no. of the study/trial: N/A.

Animal studies: N/A.

## Supporting information


Video S1

Video S2

Video S3

Video S4

Video S5

Data S1
