# Supplementary material for: Development of a system to support information sharing for medical staff in the hybrid emergency room
Source: Acute Med Surg. 2024 Sep 20;11(1):e70006. doi: 10.1002/ams2.70006 (PMC11415235; doi:10.1002/ams2.70006)
Supplement: Supplementary file 6 — Data S1 [file AMS2-11-e70006-s003.docx]

**Supporting Information Legend**

**Supp Info. 1** The video of the standard monitoring mode. The video shows the standard monitoring mode of this system and biological information monitor in fast forward. The trends of vital signs and BGA were displayed, the results of BGA and laboratory data were displayed on the screen.

**Supp Info. 2** The video with audio explaining the monitor of this system with read out the results of BGA and voice alerts. The results of BGA are revealed, the results are promptly displayed and read out. Abnormal vital signs are notified to the medical staff by reading the specific values.

**Supp Info. 3** The video of the critical care mode. The video shows the critical care mode of this system in fast forward. This mode has the function to count up the times from the start of the monitoring, and from the surgery. The trend of the laboratory data reveals fibrinogen, platelet count, hemoglobin and lactate.

**Supp Info. 4** The video of the critical care mode. The video shows the critical care mode of this system in fast forward. This mode has the function to display the icon of transfusion in the display, and count total transfusion administered.

**Supp Info. 5** The video of this monitoring system, biological information monitor and the video in the hybrid emergency room with audio. This video reveals the initial treatment of trauma patients in the hybrid emergency room with this system.
